# Retroactive Streaming Fails to Improve Concurrent Vowel Identification

**DOI:** 10.1371/journal.pone.0140466

**Published:** 2015-10-09

**Authors:** Eugene J. Brandewie, Andrew J. Oxenham

**Affiliations:** Department of Psychology, University of Minnesota, Minneapolis, Minnesota, United States of America; University of Salamanca- Institute for Neuroscience of Castille and Leon and Medical School, SPAIN

## Abstract

The sequential organization of sound over time can interact with the concurrent organization of sounds across frequency. Previous studies using simple acoustic stimuli have suggested that sequential streaming cues can retroactively affect the perceptual organization of sounds that have already occurred. It is unknown whether such effects generalize to the perception of speech sounds. Listeners’ ability to identify two simultaneously presented vowels was measured in the following conditions: no context, a preceding context stream (precursors), and a following context stream (postcursors). The context stream was comprised of brief repetitions of one of the two vowels, and the primary measure of performance was listeners’ ability to identify the other vowel. Results in the precursor condition showed a significant advantage for the identification of the second vowel compared to the no-context condition, suggesting that sequential grouping mechanisms aided the segregation of the concurrent vowels, in agreement with previous work. However, performance in the postcursor condition was significantly worse compared to the no-context condition, providing no evidence for an effect of stream segregation, and suggesting a possible interference effect. Two additional experiments involving inharmonic (jittered) vowels were performed to provide additional cues to aid retroactive stream segregation; however, neither manipulation enabled listeners to improve their identification of the target vowel. Taken together with earlier studies, the results suggest that retroactive streaming may require large spectral differences between concurrent sources and thus may not provide a robust segregation cue for natural broadband sounds such as speech.

## Introduction

Sounds that occur at the same time can originate from different sources, such as two people making an utterance at the same time. The auditory system makes use of several acoustic cues to disentangle concurrent sources. These cues include differences in fundamental frequency (F0) [1–4], differences in harmonicity [5–7], and onset and offset asynchronies [7–9]. Aside from segregating simultaneous sources, the auditory system must also follow one source (such as one person talking) over time, and distinguish it from other potentially interfering sources (such as another person talking). Cues that facilitate sequential source segregation include differences in spectral content [10], F0 [11,12], and spatial location [13].

Although the perceptual organization of concurrent and sequential sound elements are often studied separately, they clearly interact in real acoustic environments where sounds both overlap and unfold over time. To demonstrate this interaction, Darwin *et al*. [14] used a pitch-matching experiment, where a harmonic complex was presented with a single mistuned component. When the amount of mistuning was relatively small, the mistuned component had the effect of altering the overall pitch of the complex, but when the mistuning was too great, the mistuned component was heard separately and no longer influenced the overall pitch of the complex tone. However, when the harmonic complex was preceded by a stream of four brief presentations of the mistuned component in rapid succession, the influence of the mistuned harmonic on the overall pitch of the complex was greatly reduced. This effect could be explained in terms of sequential grouping mechanisms, such that the preceding components (or precursors) captured the mistuned harmonic within one stream and reduced its contribution to the simultaneous complex. A similar effect has been reported in a vowel identification task [15]. The inclusion or exclusion of a specific harmonic component can alter the perceptual identity of the vowel [8]. However, when the vowel complex was preceded and followed by repetitions of the harmonic component, the component had a reduced influence on the perception of the vowel complex, and was instead captured by the tone sequence [15]. These studies provide evidence of sequential grouping constraints on concurrent perceptual grouping.

One alternative explanation of these effects involves peripheral adaptation, beginning in the auditory nerve [16]. Peripheral adaptation is a reduction in the neural response to a stimulus, based on previous stimulation. The argument is that the preceding sequential presentations of the target component reduce the response to that component during the harmonic complex, thereby reducing its influence on the overall complex. Both Darwin *et al*. [14] and Shinn-Cunningham *et al*. [15] provided evidence that their results could not be accounted for by adaptation, based on the fact that similar effects were not achieved by simply reducing the amplitude of the target component.

A stronger test of the effects of adaptation was provided by Dau *et al*. [17] using a different paradigm. They measured detection thresholds for a pure tone embedded in a narrowband-noise masker with and without the presence of flanking noise bands that were either independent or were co-modulated in amplitude with the masker band. When the flanking bands were co-modulated with the masker, detection thresholds were lower than when the flanking bands were absent, in line with expectations based on co-modulation masking release (CMR; [18]). However, CMR was eliminated by introducing a sequence of noise bands at the frequencies of the flanking bands following the presentation of the target and masker. These ‘postcursors’ were thought to perceptually capture the flanking bands, and hence eliminate CMR, which is believed to depend on the perceptual grouping of the masker and flanking bands. These results could not be explained by adaptation, because the noise-band sequence followed the target and masker in time. Therefore any physiological reduction in stimulus response due to adaptation would occur after the target had already been presented and could therefore not affect the peripheral physiological response to the target directly. Additionally, the perceptual capture of the flankers demonstrates a process of retroactive stream segregation, in which the formation of a perceptual stream can affect the processing of an acoustic component at the beginning of the stream, before the stream has fully “built up” [19].

A number of studies have used the paradigm of presenting two concurrent synthetic vowels with equal duration to study the efficacy of different concurrent segregation cues. For example, a difference in F0 between two vowels has consistently been shown to produce an improvement in listeners’ ability to identify them both [1–3]. In contrast, differences in lateralization using interaural time differences (ITD) [20,21], or differences in amplitude- or frequency-modulation coherence [22], between the two vowels have been shown to be largely ineffective as concurrent segregation cues.

Summerfield and Assmann [2] used a double-vowel paradigm to study the influence of various types of precursors on concurrent vowel identification. These authors presented a preceding 1-s vowel sound that had the same spectrum as one of the two vowels of the brief (200-ms) double-vowel target. They found a significant improvement in identification of the second vowel in the target when the precursor was presented in the same ear (ipsilateral) as the target; however, there was no effect when the precursor was presented to the opposite ear (contralateral) or presented as an orthographic visual stimulus. These results ruled out any effect of prior knowledge of the phonemic identity, since both contralateral and visual precursors would have provided such information but did not lead to improved vowel identification. The lack of effect in the contralateral condition also ruled out any influence of prior knowledge of the spectrum of one vowel to derive the spectrum of the second vowel.

Both adaptation and sequential streaming may have contributed to the effects observed by Summerfield and Assmann [2]. One way to reduce the potential effects of adaptation, while encouraging sequential segregation, is to present the precursor as a series of short bursts, separated by silences [14,15]. A more definitive way to rule out the influence of adaptation is to present the streaming cues after the target in time, thereby inducing retroactive streaming. Most of the previous studies examining the effects of sequential grouping on concurrent perception have tested the influence of a single tone in a harmonic complex [4,8,9,14,15] or the effects of a complex sound on the perception of a single component [17,23,24]. To our knowledge, the case where both sounds have a broad spectrum, more similar to sounds in the natural environment, has not been studied. In addition, no study has used stimuli with overlapping spectra between the sequential and concurrent components. The only prior research demonstrating retroactive streaming effects on concurrent presentations [17] has used narrowband targets and maskers that were separated in frequency and thus activated separate frequency channels. To investigate the influence of retroactive streaming on concurrent sound organization, this study used vowel stimuli, which were broadband and had overlapping spectral envelopes.

The aim of the present study was to assess the influence of sequential grouping cues on the concurrent organization of broadband sounds with overlapping spectra. Measures of concurrent vowel identification were made using the double-vowel paradigm to assess successful segregation. Sequential grouping was manipulated by presenting repeated bursts of one vowel either prior to, or following, the simultaneous presentation of both vowels. To our knowledge, this study represents the first test of whether the retroactive streaming observed with narrowband or spectrally sparse stimuli [17] generalizes to more natural broadband sounds with overlapping spectra.

## Experiment 1: Effects of Sequential Streaming on Vowel Identification

### Methods

#### Listeners

Ten listeners (six female) with ages between 20–40 years participated in this experiment. All had normal hearing as verified by audiometric screening at 20 dB hearing level (HL) or better at octave frequencies between 250 and 8000 Hz. All listeners spoke American English as a first language. All listeners provided written informed consent prior to testing and were paid for their participation. The experimental protocols were approved by the Institutional Review Board of the University of Minnesota.

#### Stimuli

Five steady-state approximations of American English vowels /i/, /ɑ/, /ʊ/, /ɛ/, and /ɜ/ were synthesized at an F0 of 130 Hz using an implementation of Klatt’s vowel synthesizer [25]. The input waveform for the synthesized vowels was a harmonic complex consisting of the F0 (130 Hz) and the first 59 harmonics, all with zero phase and equal amplitude. The formant frequencies for each synthesized vowel were set by the average male formant frequencies shown by Hillenbrand *et al* [26] and are presented in Table 1. The fifth formant frequency was set at 3850 Hz for all vowels, matching the value used in the study by Summerfield and Assmann [2]. As shown in Table 1, the same formant bandwidths were used for all vowels. Each synthetic vowel had a duration of 200 ms, including 10-ms cosine-squared onset and offset ramps. No attempt was made to equalize level across the vowels after vowel simulation, leaving natural variations in level intact. The final output level for each vowel is listed in Table 1. The combined vowel stimuli ranged in level from 63.4 to 66.5 dB sound pressure level (SPL).

**Table 1:** Synthetic Vowel Properties.

		F1	F2	F3	F4	F5	Level
/i/	‘heed’	342	2322	3000	3657	3850	59.3
/ɑ/	‘hod’	652	997	2538	3486	3850	57.2
/ʊ/	‘hood’	469	1122	2434	3400	3850	61.5
/ɛ/	‘head’	588	1952	2601	3624	3850	58.8
/ɜ/	‘herd’	474	1379	1710	3334	3850	57.6
Bandwidth (Hz)		90	110	170	250	300	

Frequency (Hz) and 3-dB bandwidths of each vowel formant. The example words presented to listeners on response feedback buttons are listed in the second column. The RMS level (dB SPL) is also shown for each individual vowel in the last column.

#### Conditions

**Fig 1:**
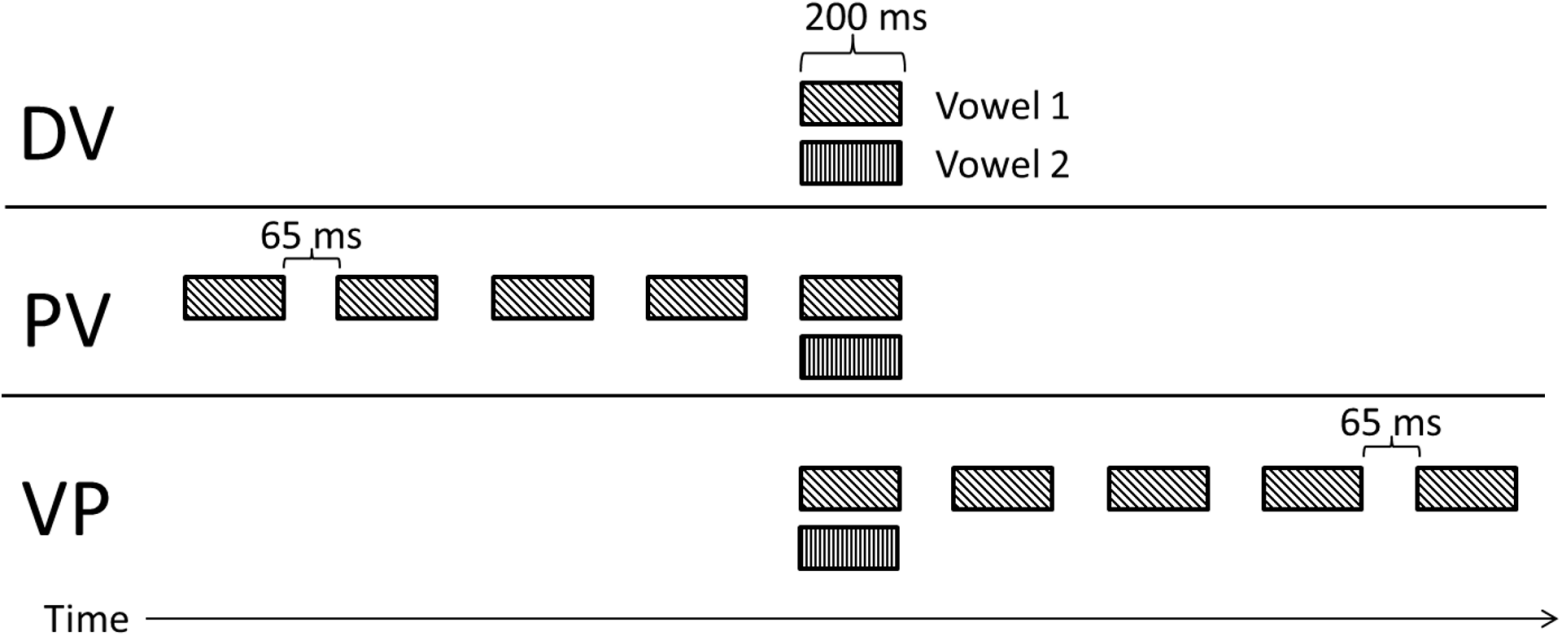
Illustration of the three context conditions for Experiment I. The Double Vowel (DV) control condition presented two simultaneous vowels in isolation. The Precursor Vowel (PV) condition presented one of the two target vowels four times prior to the simultaneous presentation. The Vowel Postcursor (VP) was the temporal reverse of the PV condition.

Listeners were tested with the double-vowel paradigm in three experimental conditions: control, precursor, and postcursor. These conditions varied in the sequential context surrounding the presentation of the two concurrently presented target vowels. Fig 1 illustrates the sequential organization of each condition. All conditions had 500 ms silence preceding and following each stimulus presentation.

The Double Vowel (DV) control condition consisted of only a single concurrent presentation of two target vowels with equal duration, synchronous onset and offset times, and an identical F0.

The Precursor Vowel (PV) condition was intended to address whether forward sequential streaming can enhance double-vowel perception by aiding object formation and segregation mechanisms without the benefit of differences in F0. Four presentations of one of the vowels were presented prior to the concurrent vowel target. The concurrent vowels themselves were identical in both the control and precursor conditions. Each presentation of the vowel was separated by a silent interval of 65 ms. This is in contrast to the precursor used by Summerfield and Assmann [2], in which the only gap between the precursor and the double-vowel was that produced by their 10.7-ms onset and offset ramps. The rhythmic presentation of multiple precursor stimuli should encourage sequential streaming, while the longer gaps should lessen the influence of peripheral neural adaptation [23,27].

The Vowel Postcursor (VP) condition is intended to test whether retroactive streaming effects can also enhance vowel perception in a way similar to the retroactive effects demonstrated by Dau *et al* [17]. This condition was a temporal inversion of the PV condition, consisting of the concurrent DV targets followed by four presentations of one of the two target vowels (separated by 65-ms silent gaps). Presenting the additional vowels after the target also helps to rule out possible adaptation effects.

#### Procedure

Listeners were instructed to report both vowels presented during the concurrent double-vowel target. Listeners responded on a graphical user interface (GUI) featuring five buttons (one for each vowel). The labels for the GUI buttons featured words for each vowel in a /hVd/ structure as shown in Table 1 (“heed”, “hod”, “hood”, “head”, “herd”). The placement of the vowels on the GUI buttons was randomized on each trial to maintain the attention of the listeners. After the listener selected two buttons, the next trial began automatically. Feedback for correct and incorrect responses was provided by highlighting the buttons of both correct vowels after each selection of two vowels. All stimulus presentation and experimental control was performed using custom software written for a MATLAB® environment. Stimuli were presented diotically at a moderate level (63.4 to 66.5 dB SPL rms, 72 dB SPL peak) over equalized headphones (Sennheiser HD 650) within a double-walled sound-attenuating chamber.

Each block of trials consisted of 20 randomized trials (one for each combination of the five vowels, excluding pairings of the same vowel). The experimental condition was fixed across a block of trials. The order of the blocks was randomized for each listener. Each listener completed 30 blocks of trials (ten per experimental condition) over the course of multiple two-hour sessions.

Prior to participation in these experiments, all listeners performed a preliminary test block to ensure they could accurately identify each of the five vowels in isolation. The preliminary test block consisted of 50 trials (5 vowels x 10 repetitions) in which single vowels were presented and listeners only reported one vowel per trial. All listeners scored at least 90% correct vowel identification (mean = 97.8%) in this preliminary test.

#### Scoring

Performance in the experimental conditions (PV, VP) was scored by measuring performance only in the “other” vowel that was not presented in the pre- or postcursors. In order to fairly compare performance in these conditions with the DV condition, performance in the DV condition was scored twice, with the first and then the second vowel being considered the “other” vowel. A trial was considered correct if either response matched the “other” vowel, and so a trial where the listener correctly identified both vowels as correct would be counted as two correct, whereas a trial where only one vowel was correctly reported was counted as one correct and one incorrect. Therefore single-vowel performance in the DV condition (DV[1]) has twice as many “trials” as the other conditions. This process is identical to the scoring method used by Summerfield and Assmann [2] in a similar paradigm. All comparisons are made with the single-vowel performance of the control condition (DV[1]). The results of the both vowels correct (DV) are presented only for comparison with previous research.

### Results and Discussion

**Fig 2:**
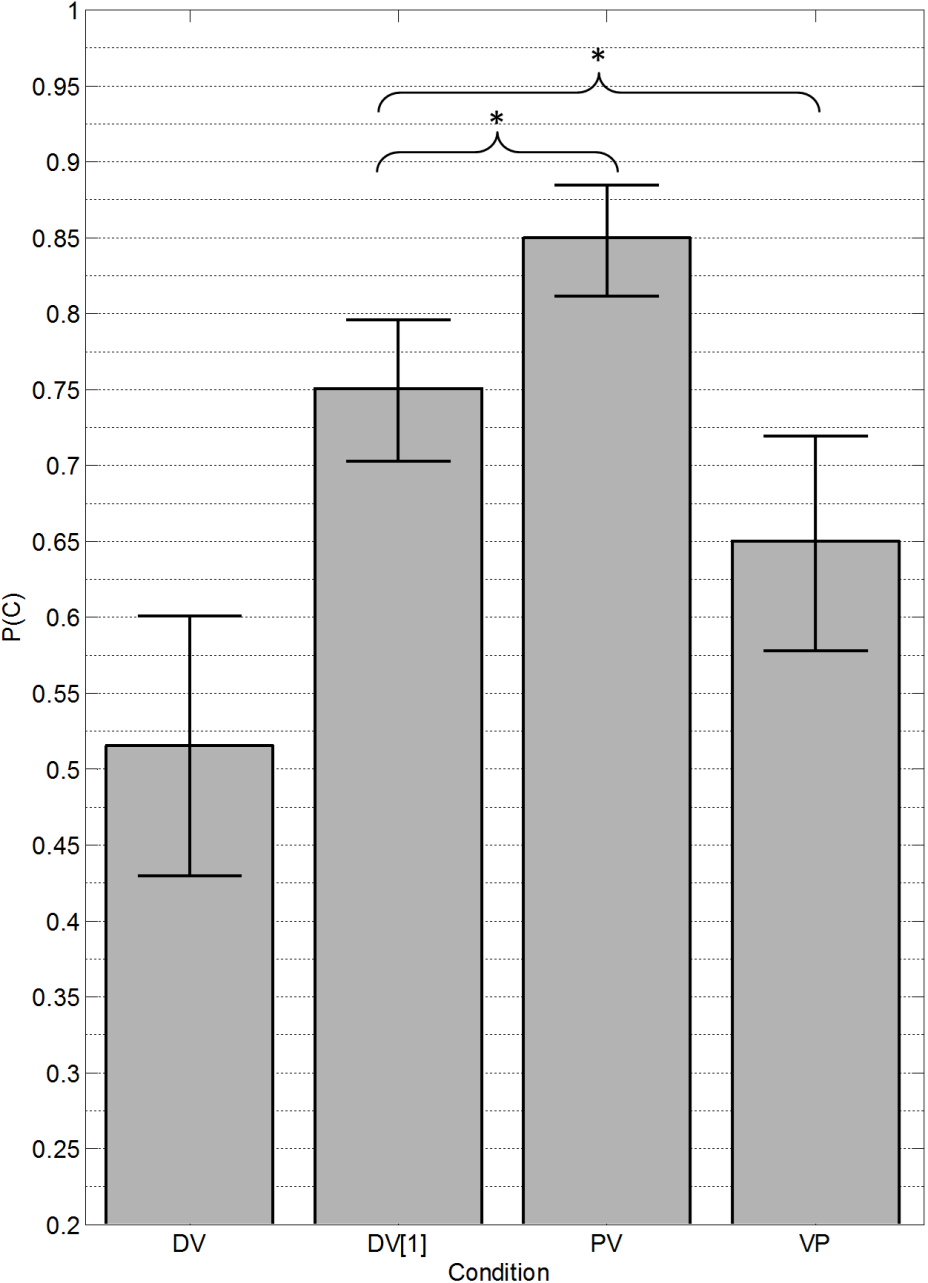
Results from Experiment 1. Mean (*n* = 10) proportion of trials in which both vowels were correctly reported (DV) or in which the single target vowel was correctly reported (DV[1], PV, VP). DV: double-vowel condition; DV[1]: double-vowel condition, but with trials scored for each target vowel separately (see Scoring section for details); PV: condition with one of the vowels presented as a precursor; VP: condition with one of the vowels presented as a postcursor. Error bars represent the standard error of the mean. An asterisk (*) indicates a statistically significant difference (*p* < 0.05).

The proportion of correct (PC) responses for each condition and each listener were converted to rationalized arcsine units (RAU) [28] for statistical analysis, but are shown as raw proportion correct in the figures. Fig 2 presents the mean proportion correct for each condition with error bars representing the standard error of the mean. For comparison, chance performance in this task is 5% (1/20) for the DV condition and 25% (1/4) for all other conditions presented in Fig 2. A repeated-measures (within-subjects) analysis of variance including the DV1, PV, and VP scores confirmed a significant main effect of condition [*F*(2,29) = 32.3, *p* < 0.001, *partial η* ^*2*^ = 0.52]. Post hoc comparisons (with Holm-Bonferroni corrections [29]) indicated that mean performance in the precursor (PV) condition (*M* = 86.0, *SD* = 11.0) was significantly higher than in the control condition (DV1) (*M* = 74.4, *SD* = 11.6), [*t*(9) = 6.09, *p* < 0.001, Cohen’s *d* = 1.03]. Performance in the postcursor (VP) condition (*M* = 64.2, *SD* = 16.0), however, was significantly lower than in the control condition (DV1) [*t*(9) = 3.97, p < 0.005, Cohen’s *d* = 0.73].

The improvement shown with the precursor suggests that sequential grouping mechanisms may have aided performance. The results are similar to and extend those of Summerfield and Assmann [2] for their ipsilateral precursor. The inclusion of the 65-ms gap between the last presentation of the precursor and the concurrent vowel pair reduces the potential influence of peripheral adaptation. Although it has been shown that adaptation can take up to 100 ms to fully recover, the 65 ms gap would likely provide ample time to regain most neural sensitivity [30]. Therefore the improvement seen with the precursor can more likely be attributed to sequential grouping cues.

The postcursor condition (VP) was expected to provide a measure of the effects of sequential streaming retroactively, without any possible influence of adaptation; yet no benefit of the postcursors was observed. An earlier streaming study [17] found strong effects of postcursors when the postcursors were spectrally remote from the target; however, no such effects were observed when the postcursors fell within the same frequency region as the target. Therefore, the fact that we did not observe a beneficial effect of the postcursors may be due to spectral overlap between the vowel in the postcursor and the vowel in the target. Indeed, because the two vowels were presented on the same F0, all components in the postcursor vowel were also present in the target vowel. The fact that performance was actually poorer with the postcursors than without suggests possible interference effects, perhaps involving auditory working memory. Similar effects have been termed “backward recognition masking” [31], where the presentation of a second sound interferes with the recognition of a sound presented earlier in time. This possibility is discussed in the final discussion section.

The following two experiments pursue the question of whether streaming based on postcursors could be beneficial in segregating sounds with overlapping spectral envelopes by manipulating differences in the spectral fine structure between the two vowels.

## Experiment 2: Effects of Harmonic Jittering

The results of Experiment 1 suggest that retroactive stream segregation may not be possible under the conditions tested, perhaps because both vowels in the task stimulated the same peripheral auditory channels. To address the question of whether sequential streaming can affect the simultaneous segregation of two stimuli with overlapping spectra, a manipulation was required that altered the spectral fine structure of the two vowels, without providing an overall segregation cue, such as an F0 difference. We achieved this by introducing a random frequency jitter to each of the frequency components of each vowel independently. This manipulation creates the opportunity for individual harmonics from the two vowels to be separately represented (or resolved) within the peripheral auditory system, without providing a reliable acoustic cue for segregating the two vowels. Our hypothesis was that the differences in the spectral fine structure of two vowels should not allow for segregation of the concurrent vowels by themselves, but may allow for segregation in the presence of sequential streaming cues, based on spectral continuity of the components in the precursor or postcursor.

### Methods

The same participants were used as in Experiment 1. The stimuli and procedures were also the same, with the exception that the frequency components of each vowel were jittered independently between trials to produce inharmonic complexes. The frequency of any given tone in the complex is given by the following equation:

$$f_{n}=F0\left(n+cr_{n}\right)$$ (1)

where F0 is the fundamental frequency (130 Hz), *n* is the harmonic rank, *c* is the jitter constant (0.3), and *r*_*n*_ is a random number with a uniform distribution between -1 and 1. This has the effect of altering the frequency of each harmonic by up to +/- 30% of the F0 from its nominal frequency. This process removes the harmonic relationship between components in each complex, but maintains the average spectral density of the components. In addition, it means that corresponding harmonics from the vowels will almost never be exactly coincident. The percept induced by these stimuli is a multitude of individual tones that is more characteristic of noisy or whispered speech, rather than a clear distinctive pitch that binds the elements together.

Participants ran in three experimental conditions matching those used in Experiment 1: jittered isolated double-vowel (JDV), jittered double-vowel with precursor (JPV), and jittered double-vowel with postcursor (JVP). In the conditions with pre- or postcursors, the same random frequency components were repeated for each presentation of the vowel within a single trial. On each new trial, a new set of random components was selected for each vowel.

### Results and Discussion

**Fig 3:**
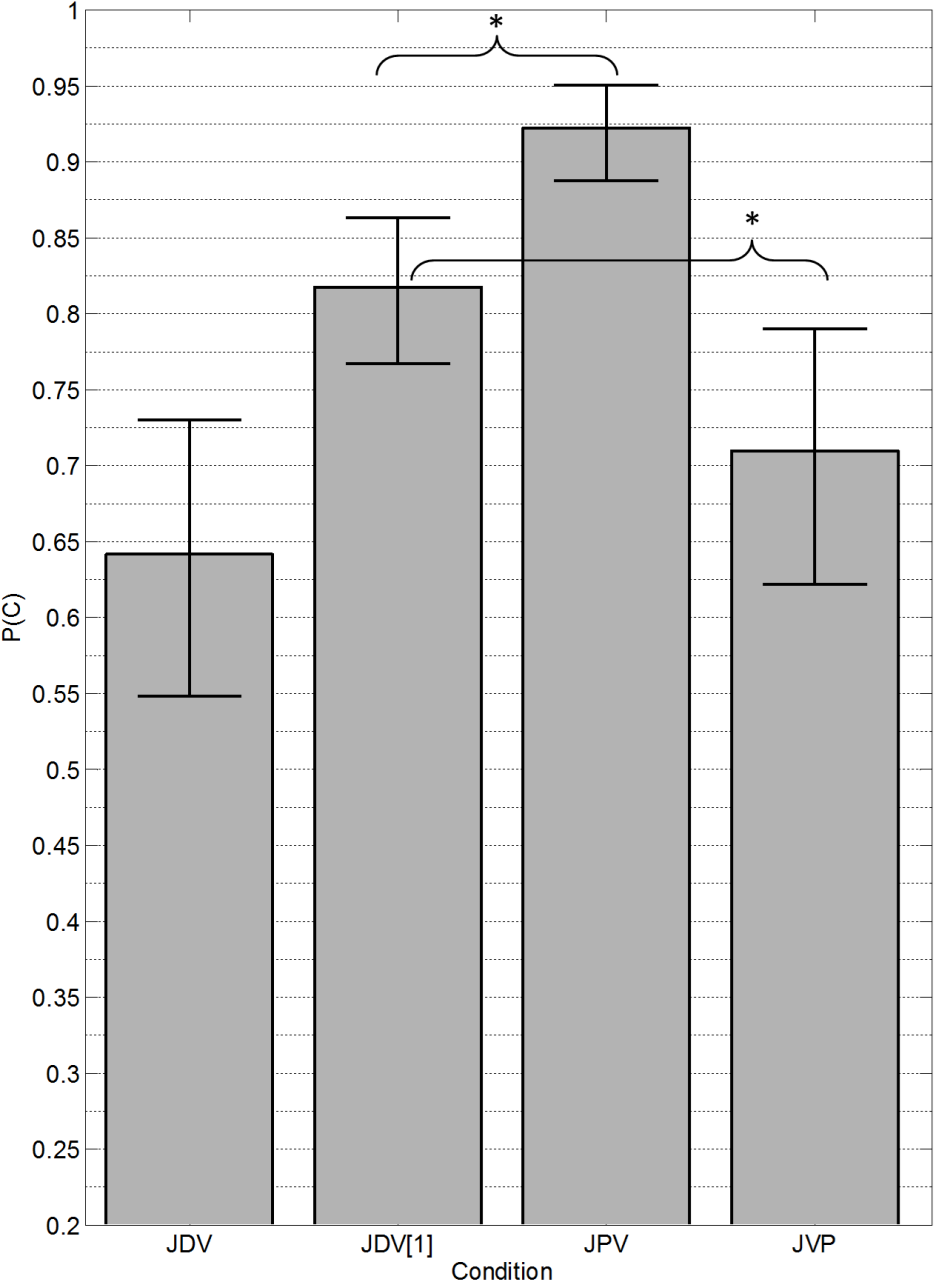
Results from Experiment 2. Mean (*n* = 10) proportion of trials in which both vowels were correctly reported (JDV) or in which the single target vowel was correctly reported (JDV[1], JPV, JVP). The conditions are the same as those shown in Fig 2, with the exception that the harmonics were jittered in frequency. Error bars represent the standard error of the mean. An asterisk (*) indicates a statistically significant difference (*p* < 0.05).

The transformation and analysis of the data were the same as for Experiment 1. Fig 3 presents the mean proportion correct for each condition with standard error bars. A within-subjects analysis of variance confirmed a significant main effect of condition [*F*(2,29) = 48.0, *p* < 0.001, *partial η* ^*2*^ = 0.53]. Post hoc comparisons indicated that performance in the precursor (JPV) condition (*M* = 96.7, *SD* = 12.7) was significantly higher than in the standard (JDV1) (*M* = 82.0, *SD* = 13.4) [*t*(9) = 7.25, *p* < 0.001, Cohen’s *d* = 1.13], and that performance in the postcursor (JVP) condition (*M* = 70.1, *SD* = 20.0) was significantly lower than in the standard (JDV1) [t(9) = 3.97, p < 0.01, Cohen’s *d* = 0.695].

The pattern of results for this experiment was nearly identical to that of Experiment 1. Performance in the postcursor condition was still reduced compared to the standard condition. This suggests one of two possibilities: the jitter manipulation was not sufficient to elicit perceptual segregation between the two vowels based on the components of the postcursor capturing the components of the non-target vowel within a sequential stream, or other effects, such as some form of backward recognition masking [31], continued to degrade the identification of the target vowel in the postcursor condition to a greater extent than any benefit of segregation. In order to test the first possibility, an additional experiment was performed to provide further separation between the vowels in terms of their spectral fine structure, this time by maintaining the jittering, but also introducing an average F0 difference between the two jittered vowels. Again, because the vowels were inharmonic, it was not expected that listeners would be able to segregate them based on average F0 differences; instead, it was postulated that the differences in spectral fine structure between the two vowels may be sufficiently large to allow the perceptual streaming of the components belonging to the non-target vowel by the pre- and postcursor stimuli.

## Experiment 3: Effects of Average F0 Differences

### Methods

The same participants were used in this experiment as in Experiments 1 and 2. The methods were nearly the same as those in Experiment 2, except that in addition to the jitter of the frequency components, an additional 4-semitone difference in nominal F0 was introduced between the two vowels (J4 conditions). This manipulation provided a nominal F0 for one vowel of 130 Hz, as in the previous experiments, and a nominal F0 for the second vowel of 163.8 Hz. However, the components were also jittered in frequency by ±30% as in the previous experiment, so any effects of pitch difference between the vowels were expected to be small. Participants ran in three experimental conditions matching those used in Experiments 1 and 2: isolated double-vowel (J4DV), double-vowel with precursor (J4PV), and double-vowel with postcursor (J4VP).

### Results and Discussion

**Fig 4:**
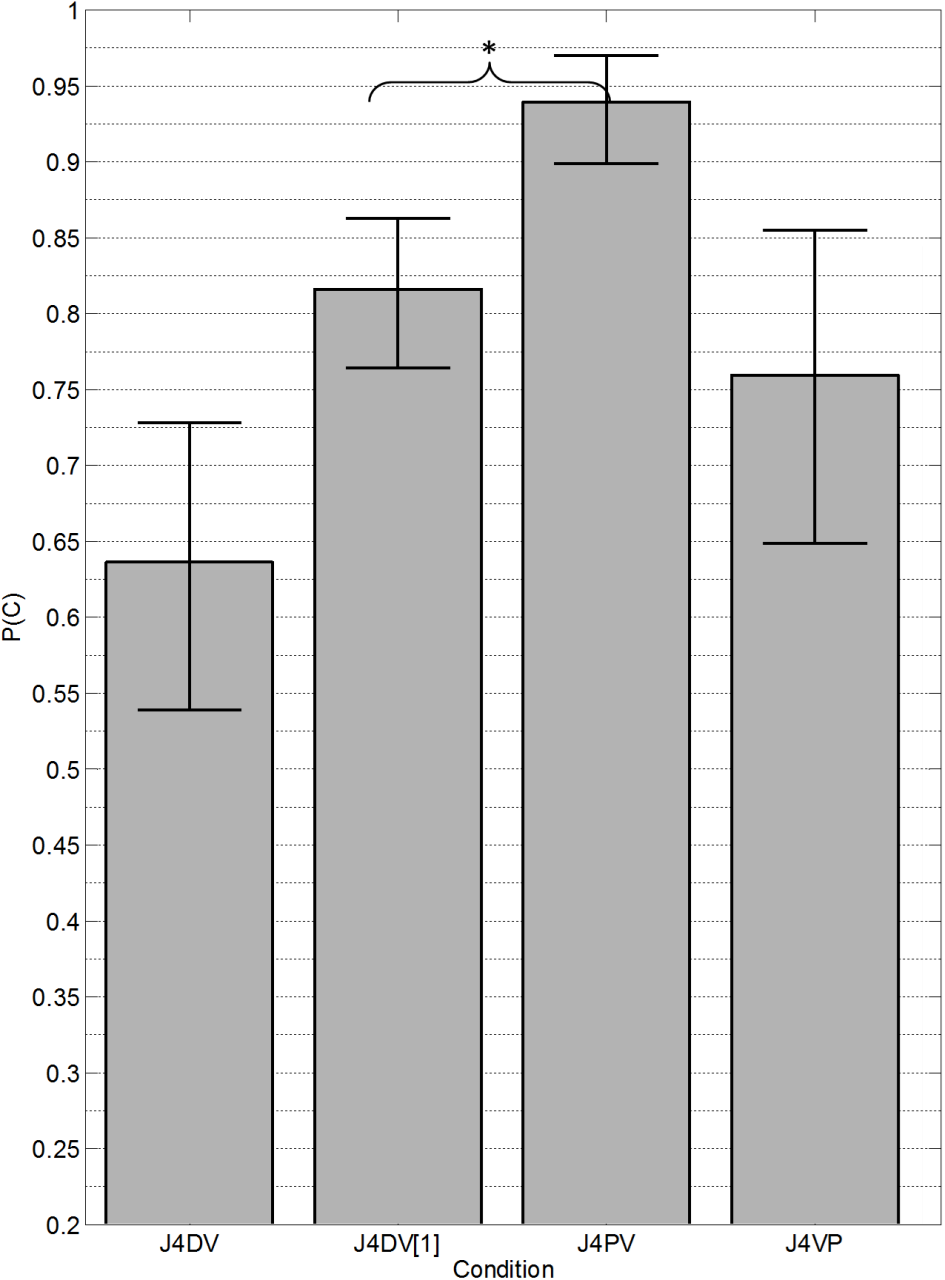
Results from Experiment 3. Mean (*n* = 10) proportion of trials in which both vowels were correctly reported (J4DV) or in which the single target vowel was correctly reported (J4DV[1], J4PV, J4VP). The conditions are the same as those shown in Fig 2, with the exception that the harmonics were jittered in frequency and the average F0 differed by 4 semitones. Error bars represent the standard error of the mean. An asterisk (*) indicates a statistically significant difference (*p* < 0.05).

The transformation and analysis of the data were performed the same way as in Experiments 1 and 2. Fig 4 presents the mean proportion correct for each condition with standard error bars. A within-subjects analysis of variance confirmed a significant main effect of condition (*F*(2,29) = 20.89, *p* < 0.001, *partial η* ^*2*^ = 0.32). Post hoc comparisons (with Holm-Bonferroni corrections) indicated that performance in the precursor (J4PV) condition (*M* = 99.9, *SD* = 16.1) was significantly higher than in the standard (J4DV1) (*M* = 81.8, *SD* = 13.7) [*t*(9) = 8.06, *p* < 0.001, Cohen’s *d* = 1.21]. Performance in the postcursor (J4VP) condition (*M* = 75.3, *SD* = 26.2) was not significantly different from that in the standard in this experiment [t(9) = 1.51, p = 0.166, Cohen’s *d* = 0.30].

The pattern of results in this experiment was similar to those in the first two experiments. The lack of a significant difference between the control and postcursor conditions in this experiment may be simply due to larger inter-subject variability in this experiment. For instance, the between-listener variance was larger in the J4VP condition (*SD* = 26.2) of this experiment than in the VP condition (*SD* = 16.0) of Experiment 1. This is also illustrated in the larger error bars in the J4VP condition in Fig 4. Visual inspection of the individual data reveals three listeners had improved performance with the postcursor, three listeners had a small decrement, and four listeners showed worse performance, as seen in previous experiments. This pattern of results might suggest that some listeners may be able to use retroactive streaming cues when enough spectral separation is provided between the two vowels. However, most listeners remained unable to benefit from the postcursors, despite the larger differences in spectral fine structure introduced by the average 4-semitone difference in F0.

## Discussion

In agreement with prior work, the control conditions in this study demonstrated performance that was significantly above chance when no segregation cues were added to distinguish the two vowels. Additionally, when precursors of one vowel were present, identification of the second vowel improved. This is consistent with, and extends the findings by, Summerfield and Assmann [2], because it suggests that the improvement stems largely from streaming processes rather than simple (peripheral) adaptation. However, such streaming processes did not seem to aid performance in the case of the postcursors, where streaming of the two vowels would have had to occur retroactively.

Early research in auditory streaming suggested that when sources are close in spectrum, or do not occupy distinct spectral regions, auditory stream segregation becomes less likely. This might help explain the lack of retroactive streaming observed in this study. Using a rapidly alternating pattern of two pure tones, classic streaming experiments [10,19] have demonstrated that a frequency difference larger than a few semitones between the tones are generally required to elicit the perception of two separate streams. Experiments 2 and 3 of this study attempted to employ both small spectral differences (via harmonic jittering) and larger differences (via jittering and a difference in mean F0) to facilitate segregation based on sequential streaming, while rendering simultaneous segregation of the two vowels unlikely due to a lack of consistent harmonicity cues. However, these manipulations did not seem to elicit segregation any more than the existing vowel spectral cues provided in the precursor condition. In the postcursor condition, retroactive streaming may have only occurred for a few listeners and only with a substantial shift in nominal F0. In all cases with the postcursors, any streaming effects may have been counteracted by perceptual interference of the postcursor vowel on the representation or storage of the target vowel.

One way to view the detrimental effect of the postcursors is by considering the top-down, schema-driven process of vowel extraction. It has been suggested that listeners choose two vowels based on those that independently best match the double-vowel spectra [2]. Another possibility is that listeners were able to develop schema, or templates, for the 20 vowel-combinations tested in this study. If this were the case, then backward recognition masking [31] by the postcursors may have interfered with phonetic processing and retrieval of this double-vowel schema. In the precursor condition of this study, it is likely that by the time the double vowel was presented, the precursor vowel had already reached a level of phonetic representation in memory such that the listener needed only to phonetically process the double-vowel without ongoing stimulation to disrupt this process. In contrast, in the postcursor condition, the presentation of the postcursor vowel may have interfered with this phonetic processing. It has been argued that acoustic representations in memory are more likely to be subject to interference by similar ongoing acoustic input [32] due to the fact that similar signals are also more likely to be perceptually grouped into the same perceptual stream. Thus ongoing speech signals, particularly those with a common F0, are more likely to interfere with the stored representation of the double-vowel in memory. It is possible that the interference effects seen in the current study relate to this constraint of working memory.

In summary, the segregation of a concurrent vowel pair was aided by the prior sequential presentation of one the vowels. Because the sequence of preceding vowels were separated by 65-ms silent gaps, the results are unlikely to be due solely to peripheral adaptation, and instead seem likely to be mediated by sequential streaming processes. However, presentation of a single-vowel stream following the concurrent vowel pair hindered identification of the target vowel and provided no evidence for the effects of retroactive sequential streaming. Instead the results demonstrated perceptual interference, even when spectral differences were introduced between the two vowels via jittering of the harmonic frequencies. The results suggest that any streaming involving retroactive components may require large spectral differences between the two concurrent sources, and may thus not play a major role in the perceptual organization of natural spectrally overlapping sounds.
